# Comparison of the Hydride-Donating Ability and Activity of Five- and Six-Membered Benzoheterocyclic Compounds in Acetonitrile

**DOI:** 10.3390/molecules27217252

**Published:** 2022-10-25

**Authors:** Jin-Ye Zhang, Xiao-Qing Zhu

**Affiliations:** The State Key Laboratory of Elemento-Organic Chemistry, College of Chemistry, Collaborative Innovation Center of Chemical Science and Engineering, Nankai University, Tianjin 300071, China

**Keywords:** five-membered benzoheterocyclic compounds, six-membered benzoheterocyclic compounds, hydride-donating ability, thermodynamic, kinetic, thermo-kinetic parameters

## Abstract

In this work, we compared the hydride-donating ability of five-membered benzoheterocyclic compounds (FMB) and six-membered benzoheterocyclic compounds (SMB), isomers of DMBI and DMIZ and of DMPZ and DMPX, using detailed thermodynamic driving forces [ΔG^o^ (XH)], kinetic intrinsic barriers (ΔG^≠^_XH/X_), and thermo-kinetic parameters [ΔG^≠^° (XH)]. For DMBI and DMIZ, the values of ΔG^o^ (XH), ΔG^≠^_XH/X_, and ΔG^≠^° (XH) are 49.2 and 53.7 kcal/mol, 35.88 and 42.04 kcal/mol, and 42.54 and 47.87 kcal/mol, respectively. For DMPZ and DMPX, the values of ΔG^o^ (XH), ΔG^≠^_XH/X_, and ΔG^≠^° (XH) are 73.2 and 79.5 kcal/mol, 35.34 and 25.02 kcal/mol, and 54.27 and 52.26 kcal/mol, respectively. It is easy to see that the FMB isomers are thermodynamically dominant and that the SMB isomers are kinetically dominant. Moreover, according to the analysis of ΔG^≠^° (XH), compared to the SMB isomers, the FMB isomers have a stronger hydride-donating ability in actual chemical reactions.

## 1. Introduction

Among the many isomeric benzoheterocyclic compounds, the most famous five-membered benzoheterocyclic compounds (FMB) are benzimidazole and benzindazole, and most famous six-membered benzoheterocyclic compounds (SMB) are quinazolinone and quinoxalinone [1,2,3,4,5,6,7,8]. The aforementioned compounds are important building blocks of many drug molecules. Among them, the core building blocks of drugs such as lansoprazole, candesartan, and albendazole are benzimidazoles [9,10,11,12,13,14]. The core building blocks of drugs such as lonidamine, benzydamine, and granisetron hydrochloride are benzidazole [15,16,17,18,19,20]. The representative drugs with a quinazolinone backbone as their core are gefitinib, afatinib, and raltitrexed [21,22,23,24,25,26]. Representative drugs with a quinoxalinone skeleton as their core are mainly used for anti-HIV treatment, and the representative drug is HBY097 [27]. In the living body, the metabolism of many drugs is carried out by donating hydride ions. Therefore, clarifying the actual hydride-donating abilities of benzimidazole, benzindazole, quinazolinone, and quinoxalinone is crucial for drug development. Careful observation of the structures of the two isomer compounds mentioned above shows that quinoxalinone and quinazolinone have one more carbonyl group than benzimidazole and benzindazole. Thus, what is the specific difference between the two groups of isomers in terms of their hydride-donating ability? Additionally, what is the effect of increasing the carbonyl groups on the hydride-donating ability of the compounds?

To answer the above questions, we chose 1,3-dimethyl-2-phenyl-2,3-dihydro-1H-benzo[d]imidazole (DMBI), 1,2-dimethyl-3-phenyl-2,3-dihydro-1H-indazole (DMIZ), 1,3-dimethyl-2-phenyl-2,3-dihydroquinazolin-4(1H)-one (DMPZ), and 1,4-dimethyl-3-phenyl-3,4-dihydroquinoxalin-2(1H)-one (DMPX) as the research subjects. In this paper, the thermodynamic driving force [ΔG^o^ (XH)], kinetic intrinsic barrier (ΔG^≠^_XH/X_), and thermo-kinetic parameter [ΔG^≠^° (XH)] are used to describe the hydride-donating ability of compounds in thermodynamic, kinetic, and actual chemical reactions [28]. ΔG^o^ (XH) and ΔG^≠^_XH/X_ are the dissociation free energy of the X-H bond and the activation free energy of the self-exchange H^−^T reaction for XH^−^ (XH^−^ + X^+^→X^+^ + XH^−^), respectively [29]. In previous work, we combined the thermodynamic driving force and kinetic intrinsic barrier to propose the concept of thermo-kinetic parameters [ΔG^≠^° (XH)] that are able to describe the hydride-donating ability of compounds in actual chemical reactions (Equations (1)–(3)): the larger the value, the weaker the hydride-donating ability of the compounds [30,31].

## 2. Results

**Scheme 1:**
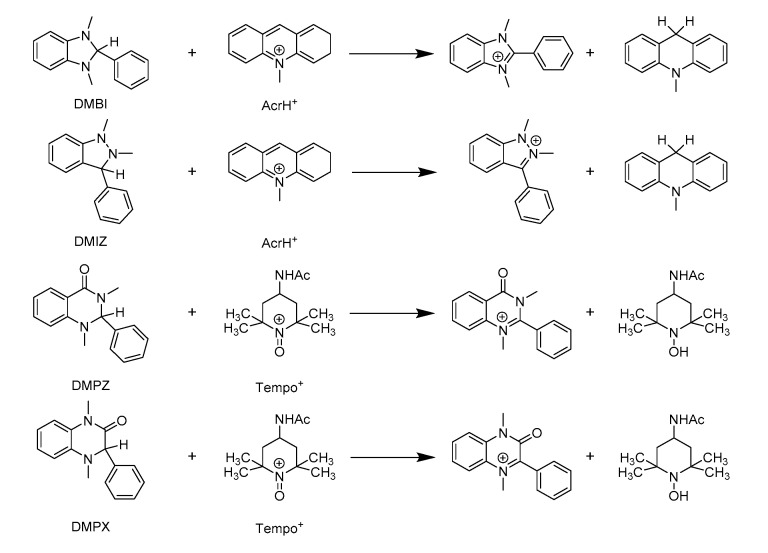
Hydride-transfer reaction equations for DMBI, DMIZ and DMPZ, DMPX isomers.

**Figure 1:**
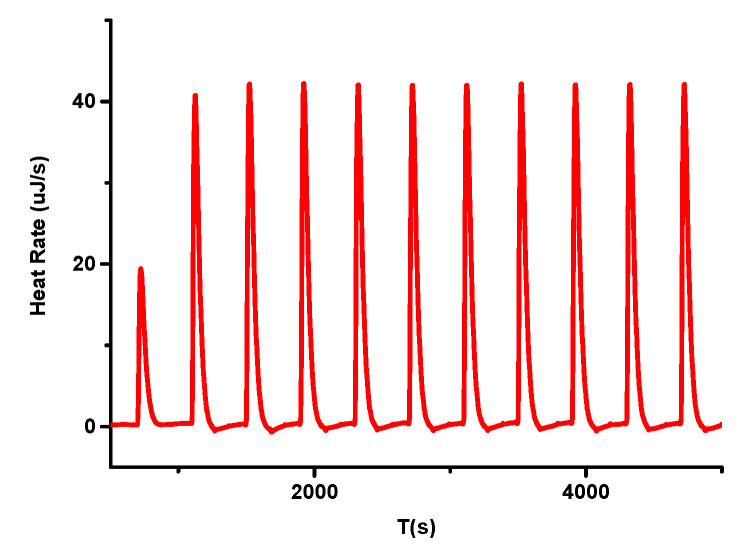
Isothermal titration calorimetry (ITC) graph of the reaction heat of DMPZ with TEMPO^+^ in acetonitrile at 298 K. Titration was conducted by adding 10 μL of DMPZ (2.0 mM) into the acetonitrile containing TEMPO^+^ (ca.10 mM) every 400 s.

**Figure 2:**
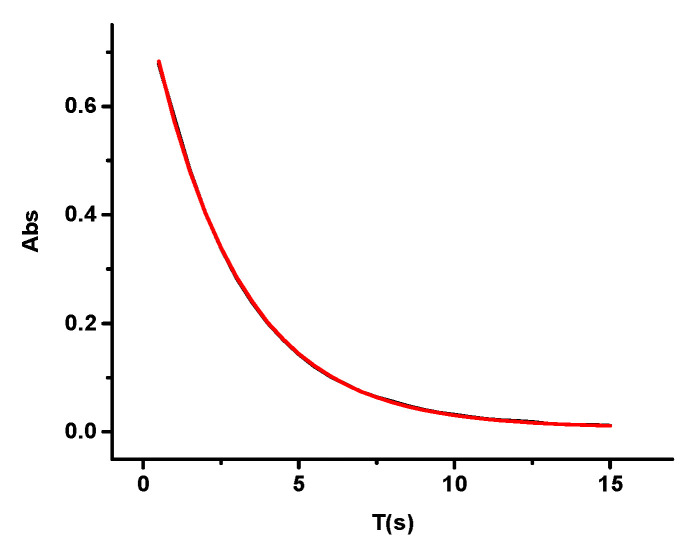
Time profile of the UV absorbance at 417 nm due to AcrH^+^ for the reactions of AcrH^+^ (0.1 mM) with DMBI (2 mM) in acetonitrile at 298 K.

Two sets of isomers, DMBI, DMIZ and DMPZ, DMPX, were synthesized by previously reported methods and identified by ^1^H and ^13^C NMR [32,33] (see Supplementary Materials). The enthalpy change and the second-rate constant of the above isomers reacting with hydride acceptors was determined in dry and anaerobic acetonitrile using CSC-4200 ITC and an Applied Photophysics SX.18MV-R stopped-flow apparatus at 298 K, respectively (Figure 1 and Figure 2) [31]. The second-rate constant (k_2_), activation free energies (ΔG^≠^_XH/Y_), and molar free energies ΔG^o^ (XH/Y) of the above reactions (Scheme 1) are listed in Table 1. The values of ΔG^o^ (XH), ΔG^≠^_XH/X_, and ΔG^≠^° (XH) were easily calculated using Table 1 data and Equations (1)–(3) (Table 2).

ΔG° = ΔG°_H−D_ (XH) + ΔG°_H−A_ (Y^+^) (1)

ΔG^≠^_XH/Y_ = ΔG^≠^° (XH) + ΔG^≠^° (Y^+^) (2)

ΔG^≠^° (XH) = 1/2 [ΔG^≠^_XH/X_ + ΔG° (XH)] (3)

## 3. Discussion

### 3.1. Analysis of Thermodynamic Driving Forces of DMBI, DMIZ, DMPZ, and DMPX as Hydride Donors in Acetonitrile

The ΔG^o^ (XH) values of DMBI, DMIZ, DMPZ, and DMPX are 49.2, 53.7, 73.2, and 79.5 kcal/mol, respectively (Table 3). The order of the hydride-donating ability of the two isomers in thermodynamics is DMPX > DMPZ > DMIZ > DMBI, which indicates that DMBI has the best hydride-donating ability. For the FMB isomers, the value of ΔG^o^ (DMBI) is 4.5 kcal/mol smaller than ΔG^o^ (DMIZ), which indicates that DMBI has better hydride-donating ability than DMIZ in thermodynamics. Additionally, for the SMB isomers, the value of ΔG^o^ (DMPZ) is 6.3 kcal/mol smaller than ΔG^o^ (DMPX), which indicates that DMPZ has better hydride-donating ability than DMPX in thermodynamics. In addition, when a carbonyl group was added to the structure of DMBI, regardless of the position of the carbonyl group, the hydride-donating ability of the changed compound was decreased in terms of its thermodynamics. The carbonyl group is an electron-withdrawing group. Due to the induction effect, the ability of DMPZ and DMPX to donate hydride ions is less than that of DMBI in terms of thermodynamics. The closer the carbonyl group is to the site where hydride ions are released, the more obvious the effect is. Obviously, the ΔG^o^ (XH) value of the five-membered compound isomers is smaller than that of the six-membered compound isomers, which means the five-membered compound isomers have a better hydride-donating ability in terms of thermodynamics.

### 3.2. Analysis of Kinetic Intrinsic Barriers of DMBI, DMIZ, DMPZ, and DMPX as Hydride Donors in Acetonitrile

According to Table 3, the ΔG^≠^_XH/X_ values of DMBI, DMIZ, DMPZ, and DMPX are 35.88, 42.04, 35.34, and 25.02 kcal/mol, respectively, and the order of ΔG^≠^_XH/X_ is DMIZ > DMBI > DMPZ > DMPX, which means that DMPX has the best hydride-donating ability in terms of kinetics. This situation is different from the order for thermodynamics. For the FMB isomers, the value of ΔG^≠^_XH/X_ (DMBI) is 6.16 kcal/mol smaller than ΔG^≠^_XH/X_ (DMIZ). This means that DMBI has a better hydride-donating ability than DMIZ in terms of kinetics, which is the same as the thermodynamic situation. Additionally, for the SMB isomers, the value of ΔG^≠^_XH/X_ (DMPX) is 10.32 kcal/mol smaller than ΔG^≠^_XH/X_ (DMPZ), which means that DMPX has a better hydride-donating ability than DMPZ in terms of kinetics, which is the opposite of the situation for thermodynamics. When a carbonyl group was added to the structure of DMBI, its kinetic intrinsic barrier decreased and the hydride-donating ability of compounds increased, regardless of the position. This situation should be related to the structure of the five-membered ring and the six-membered ring. Generally, the steric hindrance of the five-membered ring is greater than that of the six-membered ring. Besides that, the ΔG^≠^_XH/X_ value of the five-membered compound isomers is bigger than that of the six-membered compound isomers, which indicates that the six-membered compound isomers have a better hydride-donating ability in terms of kinetics. The above analysis shows that the thermodynamic and kinetic effects on the hydride-donating ability of the two groups of isomers are different. Therefore, the ability of a compound to release hydride ions cannot be analyzed by just one thermodynamic or kinetic parameter.

### 3.3. Analysis of Thermo-Kinetic Parameters of DMBI, DMIZ, DMPZ, and DMPX as Hydride Donors in Acetonitrile

The Δ*G*^≠o^ (XH) value reflects the hydride-donating ability of the compounds in an actual chemical reaction. According to Table 3, the Δ*G*^≠o^ (XH) values of DMBI, DMIZ, DMPZ, and DMPX are 42.54, 47.87, 54.27, and 52.26 kcal/mol, respectively, and the order of Δ*G*^≠o^ (XH) is DMPZ > DMPX > DMIZ > DMBI, which indicates that DMBI has the best hydride-donating ability in actual chemical reactions. For the FMB isomers, the values of ΔG^o^ (DMBI) and ΔG^≠^_XH/X_ (DMBI) are both smaller than ΔG^o^ (DMIZ) and ΔG^≠^_XH/X_ (DMIZ). According to the definition of Δ*G*^≠o^ (XH), ΔG^o^ (XH) and ΔG^≠^_XH/X_ make the same contribution to Δ*G*^≠o^ (XH) (Equation (3)). Therefore, the Δ*G*^≠o^ (DMBI) value is 5.33 kcal/mol smaller than Δ*G*^≠o^ (DMIZ), which indicates that DMBI has a better hydride-donating ability than DMIZ in actual chemical reactions. For the SMB isomers, although the ΔG^o^ (DMPZ) value is 6.3 kcal/mol smaller than ΔG^o^ (DMPX), the ΔG^≠^_XH/X_ (DMPZ) value is 10.32 larger than ΔG^≠^_XH/X_ (DMPX). Overall, the Δ*G*^≠o^ (DMPX) value is 2.01 kcal/mol smaller than Δ*G*^≠o^ (DMPZ), which indicates that DMPX has a better hydride-donating ability than DMPZ in actual chemical reactions. In addition, the Δ*G*^≠o^ (DMBI) value is smaller than both the Δ*G*^≠o^ (DMPX) and Δ*G*^≠o^ (DMPZ) values. Although the steric hindrance of the six-membered ring is smaller than that of the five-membered ring, the induction effect of the carbonyl group is significantly stronger than that of the steric hindrance. Therefore, the increase in the carbonyl group would reduce the hydride-donating ability of the compound in actual chemical reactions. In total, the order of the hydride-donating ability of compounds in the actual chemical reactions is different from the order of both thermodynamics and kinetics. Therefore, it is necessary to use thermo-kinetic parameters instead of thermodynamic driving forces and kinetic intrinsic barriers when judging the hydride-donating ability in actual chemical reactions.

## 4. Conclusions

In this paper, we compared the ability of the two isomers, FMB and SMB, to release hydride ions using thermodynamic driving forces, kinetic intrinsic barriers, and thermo-kinetic parameters. Using thermo-kinetic parameters to describe the hydride-donating ability of compounds in actual chemical reactions is more scientific and accurate than using thermodynamic driving forces or kinetic intrinsic barriers alone. In short, the FMB isomers have a stronger ability to release hydride ions than the SMB isomers in actual chemical reactions. For FMB isomers, the hydride-donating ability of DMBI is stronger than that of DMIZ, and for SMB, the hydride-donating ability of DMPX is stronger than that of DMPZ in actual chemical reactions. In addition, increasing the carbonyl groups reduces the hydride-donating ability of the compound in actual chemical reactions. We believe that this method has important guiding significance for the synthesis and screening of FMB and SMB drugs.

## Figures and Tables

**Table 1:** Second-order rate constants (*k*_2_), activation free energies (ΔG^≠^), and molar free energy change (ΔG°) values of oxidations of FMB and SMB isomers compounds in acetonitrile at 298 K.

	DMBI/DMIZ + AcrH^+^		DMPZ/DMPX + TEMPO^+^	
	DMBI	DMIZ	DMPZ	DMPX
*k*_2_ *^a^*	1.77 × 10^2^	2.17 × 10^−2^	1.68	50.27
ΔG^≠ *b*^	14.38	19.71	17.14	15.13
ΔG° *^c^*	−27.0	−22.5	−27.5	−21.2

*^a^ k*_2_ (M^−1^ s^−1^) is the second-order rate constant of the hydride transfer in acetonitrile at 298 K. The uncertainty is smaller than 5%. *^b^* Derived from Eyring equation (T = 298 K), the unit is kcal/mol. *^c^* ΔG° is equal to the corresponding reaction heat; the latter was measured by titration calorimetry in acetonitrile at 298 K. The data given in kcal/mol are the average values of at least three independent runs.

**Table 2:** Bond formation free energy and thermo-kinetic parameters of hydride acceptor used in this work (T = 298 K).

Compounds	ΔG° (Y^+^) *^a^*	ΔG^≠o^ (Y^+^) *^b^*
AcrH+	−76.2	−28.16
TEMPO+	−100.7	−37.13

*^a,b^* Reference from [34]; the unit is kcal/mol.

**Table 3:** Thermodynamic driving forces [ΔG^o^ (XH)], self-exchange reaction activation energies [ΔG^≠^_XH/X_], and thermo-kinetic parameters [ΔG^≠o^ (XH)] of DMBI, DMIZ, DMPZ, and DMPX as hydride donors in acetonitrile at 298 K.

	DMBI	DMIZ	DMPZ	DMPX
ΔG° (XH)	49.2	53.7	73.2	79.5
ΔG^≠^_XH/X_	35.88	42.04	35.34	25.02
ΔG^≠^° (XH)	42.54	47.87	54.27	52.26

The unit is kcal/mol.

## Data Availability

Not applicable.

## Supplementary Materials

The following supporting information can be downloaded at https://www.mdpi.com/article/10.3390/molecules27217252/s1: Detailed ^1^H and ^13^C NMR data of typical compounds [33,34,35,36]. The thermodynamic and kinetic test data of the compounds with different substituents and the corresponding thermodynamic driving force, kinetic intrinsic barrier and thermo-kinetic parameters values.

## Sample Availability

Samples of the compounds are available from the authors.
