# Supplementary material for: Comparison of the Hydride-Donating Ability and Activity of Five- and Six-Membered Benzoheterocyclic Compounds in Acetonitrile
Source: Molecules. 2022 Oct 25;27(21):7252. doi: 10.3390/molecules27217252 (PMC9658978; doi:10.3390/molecules27217252)
Supplement: Supplementary file 1 [file molecules-27-07252-s001.zip › molecules-1979906-supplementary.pdf]

Supporting Information

Comparison of the Hydride-Donating ability and activity of five and six-membered benzoheterocyclic compounds in acetonitrile

Jin-Ye Zhang[1,*], and Xiao-Qing Zhu[2,*]

The State Key Laboratory of Elemento-Organic Chemistry, Department of Chemistry, Collaborative Innovation Center of Chemical Science and Engineering, Nankai University, Tianjin 300071, China

*Corresponding author:

Dr. Jin-Ye Zhang, The State Key Laboratory of Elemento-Organic Chemistry, Department of Chemistry, Collaborative Innovation Center of Chemical Science and Engineering, Nankai University, Tianjin 300071, China. E-mail: zjynku@163.com

Prof. Dr. Xiao-Qing Zhu, The State Key Laboratory of Elemento-Organic Chemistry, Department of Chemistry, Collaborative Innovation Center of Chemical Science and Engineering, Nankai University, Tianjin 300071, China. E-mail: xqzhu@nankai.edu.cn

Content | Page

SI.	The Synthesis of of the Representative Compounds	S2-6
SII.	^{1}H NMR and ^{13}C NMR of the Representative Compounds	S7-10
SIII.	The Thermodynamic and Kinetic Test Data of the Compounds with Different Substituents and the Corresponding Thermodynamic Driving Force, Kinetic Intrinsic Barrier and Thermo-kinetic Parameters Values.	S10-11

SI. The Synthesis of of the Representative Compounds

Syntheses of DMBI:[1]

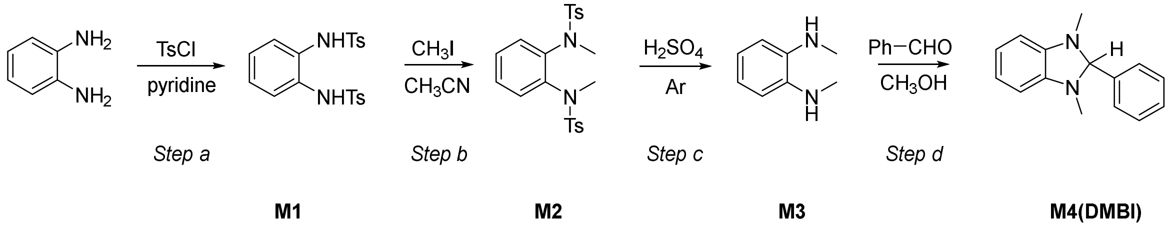

(Ts = p-toluenesulfonyl)

Step a:

A solution of o-phenylenediamine (50 mmol) in dry pyridine (50 mL) was added to a solution of p-toluenesulfonyl chloride (100 mmol) in dry pyridine (40 mL). The resulted mixture was stirred at room temperature for 18 h. By slow addition of 15% aqueous HCl, a precipitate was formed. The solid were dissolved in EtOH (140 mL) and refluxed for 4 h, and then stored in a refrigerator for crystallization. After filtration, N,N′-di(p-toluenesulufonyl)-ophenylene-diamine was obtained **(M1 yield 70%)**.

Step b:

15 g of K_2CO_3 was added to acetonitrile (200 mL) at room temperature, and after stirring for about 30 min, 25 g (0.06mol) of M1 and 21 g of CH_3I (0.24 mol) were added to the system, and the mixture was refluxed at 85°C for 8 h. After the system was cooled to room temperature, the solvent was spin-dried, extracted with water/dichloromethane, and recrystallized from ethanol to obtain white rod-shaped crystals **(M2 yield 75%)**.

Step c:

25 mL of 98% concentrated sulfuric acid was added to 20 g of M2 under argon protection, and heated at 85° C for 4 h under argon protection. After the system was cooled to room temperature, the system was poured into 100 mL of ice water, and then aqueous 4N NaOH (aq) was added until a large amount of white solids were precipitated. The residue was extracted with ether. The ether layer was washed with saturated sodium chloride solution and dried over Na_2SO_4, concentrated then dried to give crude product **(M3 yield 50%)**. This crude product was used for the following reactions without further purification.

Step d:

Add M3 and equal equivalent amount of benzaldehyde to an appropriate amount of methanol at room temperature, add about three drops of glacial acetic acid for catalysis, stir vigorously at room temperature, and a solid appears after about 30 min. Suction filtration, EtOH/H_2O recrystallization twice to obtain white solid **M4 (DMBI)**, the yield is about 80%.

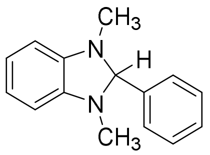

^{1}H NMR (400 MHz, $CDCl_3$) 7.40 (dd, J = 6.5, 3.0 Hz, 2H), 7.30 – 7.19 (m, 3H), 6.62 – 6.50 (m, 2H), 6.31 – 6.20 (m, 2H), 4.70 (s, 1H), 2.39 (s, 6H).

^{13}C NMR (101 MHz, $CDCl_3$) 142.09 (s), 139.07 (s), 129.33 (s), 128.86 (s), 128.47 (s), 119.30 (s), 105.71 (s), 94.02 (s), 33.16 (s).

Syntheses of DMIZ: [2]

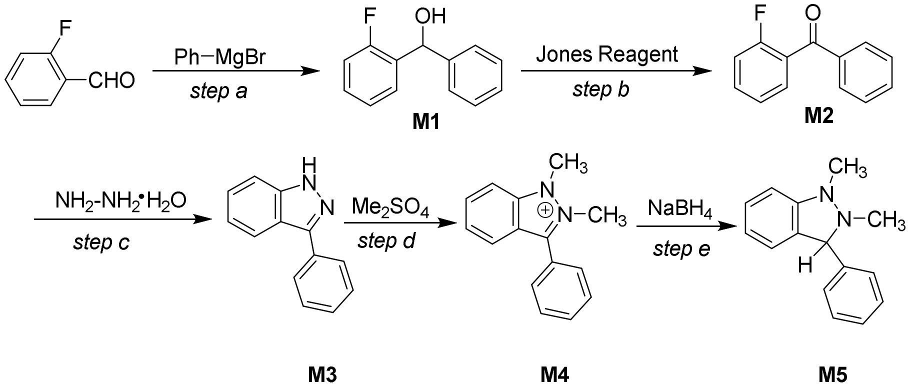

step a:

2-fluorobenzaldehyde (4.5 mmol) was dissolved in THF (50 mL), the THF solution of C_6H_5MgBr (9 mmol) was slowly dropped in the mixture. The mixture was refluxed for 4 h until the reaction finished. Then the mixture was quenched by NH_4Cl aqueous solution, and extracted by ethyl acetate, dried over $MgSO_4$. The ethyl acetate was removed to give **M1** as white solid **(yield 50%)**.

step b:

M1 (5 mmol) was dissolved in acetone (50 mL), excessive Jones reagent was added in to oxidize the **1**. After the reaction finished, the mixture was extracted by ethyl acetate, dried over $MgSO_4$. The ethyl acetate was removed to give **M2** as white solid **(yield 60%)**.

Step c:

M2 (4 mmol) was dissolved in pyridine (50 mL), hydrazine (4 mmol) was added in the mixture and stirred overnight at 110 ^{o}C. After the reaction finished, ethyl acetate (50 mL) was added in the mixture, and washed with water to remove the pyridine. The organic phase was removed to give the crude product. Purification through column chromatography will get pure products **M3 (yield 67%)**.

Step d:

M3 (4 mmol) was dissolved in dimethyl sulfate (40 mmol), and stirred overnight at 110 ^{o}C. After the reaction finished, hot water was added in to quench dimethyl sulfate. The methanol which was formed from the decomposition of dimethyl sulfate was removed from the mixture, then $HClO_4$ (aq) (70 %) was added in to produce the white precipitation. The solid was filtered, and recrystallized from ethanol to give **M4 (yield 70%)**.

Step e:

M4 (4 mmol) was dissolved in CH_3CN (30 mL), $NaBH_4$ (6 mmol) was added in and stirred for 5 h. The CH_3CN was removed by rotary evaporator, the crude product was purified through column chromatography to give final pure product **M5 (DMIZ) (yield 80%)**.

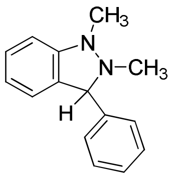

^{1}H NMR (400 MHz, $CDCl_3$) 7.36 (ddd, J = 22.7, 13.3, 7.0 Hz, 5H), 7.17 (t, J = 7.6 Hz, 1H), 6.83 – 6.64 (m, 3H), 4.75 (s, 1H), 2.95 (s, 3H), 2.66 (s, 3H).

^{13}C NMR (101 MHz, $CDCl_3$) 150.82 (s), 141.38 (s), 131.19 (s), 128.81 (s), 128.52 (s), 128.17 (s), 127.85 (s), 123.07 (s), 121.02 (s), 109.95 (s), 75.26 (s), 42.01 (s), 41.29 (s).

Syntheses of DMPZ: [3]

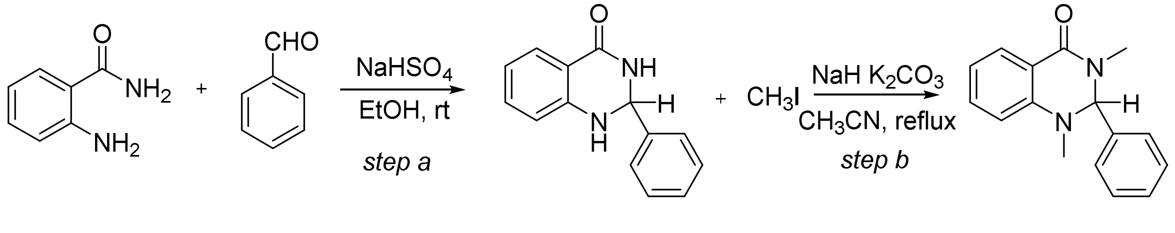

Step a:

Equivalent amounts of anthranilamide, benzaldehyde and sodium bisulfate at room temperature were added to ethanol and stirred, and the reaction was terminated after 4 h. Suction filtration to obtain white solid, ethanol recrystallization to obtain **M1**, yield 85%.

Step b:

M1 and 4 times equivalents of CH_3I, NaH and K_2CO_3 were added to acetonitrile, and the reaction was terminated after refluxing for 8 h. CH_2Cl_2/H_2O extraction, take the organic phase to spin dry and then separate by column chromatography to obtain **M2 (DMPZ)** with a yield of about 60%.

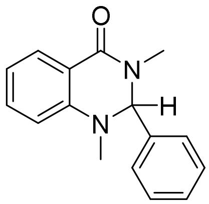

^{1}H NMR (400 MHz, $CDCl_3$) 8.02 (d, J = 7.7 Hz, 1H), 7.31 (dt, J = 13.7, 6.6 Hz, 4H), 7.21 (d, J = 7.2 Hz, 2H), 6.84 (t, J = 7.5 Hz, 1H), 6.48 (d, J = 8.2 Hz, 1H), 5.41 (s, 1H), 2.99 (s, 3H), 2.79 (s, 3H).

^{13}C NMR (101 MHz, $CDCl_3$) 163.19 (s), 146.89 (s), 137.55 (s), 134.47 (s), 129.98 (s), 129.59 (s), 129.20 (s), 127.01 (s), 118.70 (s), 116.71 (s), 112.16 (s), 81.65 (s), 36.05 (s), 33.11 (s).

Syntheses of DMPX:[4]

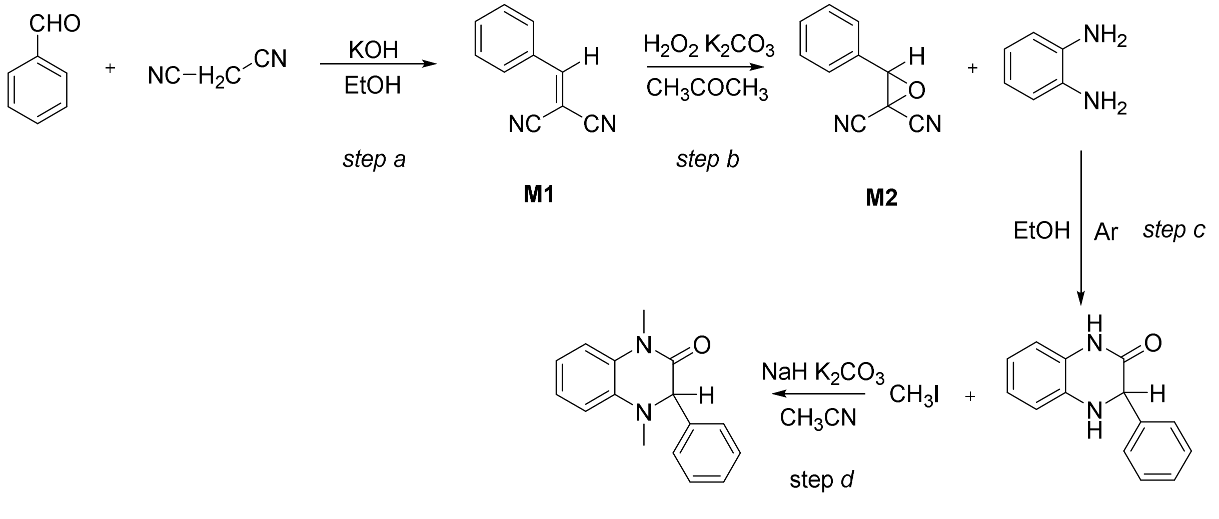

Step a:

Equal equivalents of benzaldehyde and malononitrile were added to a one-mouth flask containing ethanol (100 mL) and catalyzed by adding a few drops of 10% aqueous potassium hydroxide under an ice bath. After a few minutes, the solution became cloudy and the reaction ended after two hours. Filtration, followed by recrystallization using ethanol/water, a white solid was obtained with a yield of about 80% **(M1)**.

Step b:

1.2 g (7.78 mmol) M1 was dissolved into acetone (10 mL), and an equivalent 1 mol/L of Na_2CO_3 (aq) was added and stirred in an ice bath for 15 min. An equivalent aqueous solution of H_2O_2 (30 wt%) diluted with acetone was then added, and the reaction was terminated after about 4 h. The reaction solution was spun dry, and the white solid was obtained by column chromatography with a yield of about 80% **(M2)**.

Step c:

Equal equivalent amounts of M2 and o-phenylenediamine were added to ethanol , and the reaction was terminated by heating reflux under argon protection for 6 h. The solution was spun dry and washed with a large amount of diethyl ether to obtain a dark red solid with a yield of about 45% **(M3)**.

Step d:

M1 and 4 times equivalents of CH_3I, NaH and K_2CO_3 were added to acetonitrile, and the reaction was terminated after refluxing for 8 h. CH_2Cl_2/H_2O extraction, take the organic phase to spin dry and then separate by column chromatography to obtain **M4 (DMPX)** with a yield of about 60%.

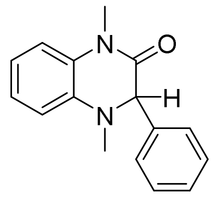

^{1}H NMR (400 MHz, $CDCl_3$) 7.31 – 7.07 (m), 6.99 (dd, *J* = 7.9, 1.3 Hz), 6.90 (td, *J* = 7.8, 1.2 Hz), 6.72 (d, *J* = 8.0 Hz), 5.03 (s, 1H), 3.45 (s, 3H), 2.91 (s, 3H)
^{13}C NMR (101 MHz, $CDCl_3$) 166.34 (s), 137.43 (s), 136.95 (s), 129.42 (s), 129.38 (s), 129.00 (s), 127.69 (s), 124.97 (s), 119.13 (s), 115.09 (s), 112.09 (s), 68.72 (s), 36.77 (s), 29.89 (s).

References

1. Zhu, X. Q.; Deng, F. H.; Yang, J. D.; Li, X. T.; Chen, Q.; Lei, N. P.; Meng, F. K.; Zhao, X. P.; Han, S. H.; Hao, E. J.; Mu, Y. Y., A classical but new kinetic equation for hydride transfer reactions. *Org Biomol Chem* **2013,** *11* (36), 6071-89.
2. Shen, G. B.; Xia, K.; Li, X. T.; Li, J. L.; Fu, Y. H.; Yuan, L.; Zhu, X. Q., Prediction of Kinetic Isotope Effects for Various Hydride Transfer Reactions Using a New Kinetic Model. *J Phys Chem A* **2016,** *120* (11), 1779-99.
3. Yoshiaki Nakao, H. I., Kyalo Stephen Kanyiva, and Tamejiro Hiyama, Direct Alkenylation and Alkylation of Pyridone Derivatives by Ni/AlMe3 Catalysis. *J. Am. Chem. Soc.* **2009,** *131*, 15996–15997.
4. Edward C. Taylor, C. A. M., and Jerauld S. Skotnicki, Heterocyclization with cyano and sulfonyl epoxides. Preparation of quinoxalines and tetrahydroquinoxalines. *J. Org. Chem.* **1980,** *45*, 2512-2515.

SII. ^{1}H NMR and ^{13}C NMR of Representative Compounds

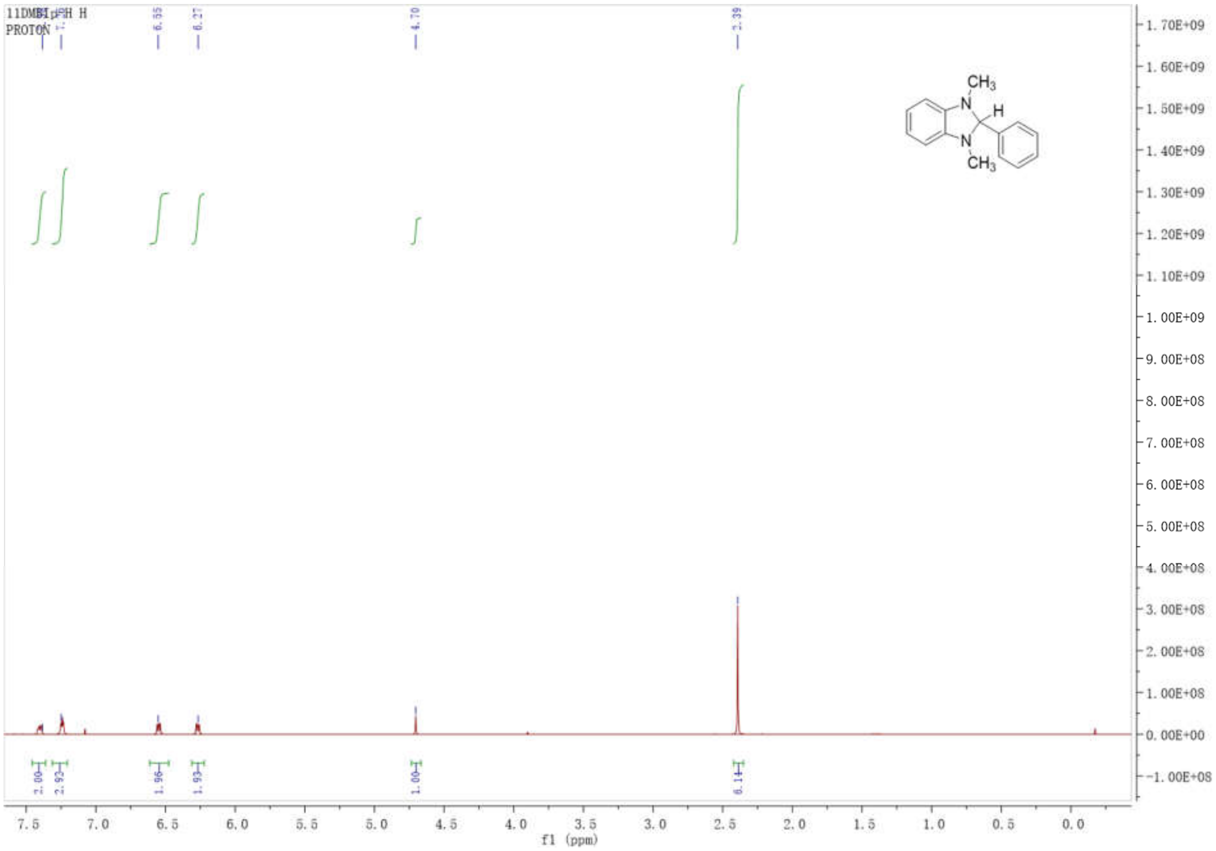

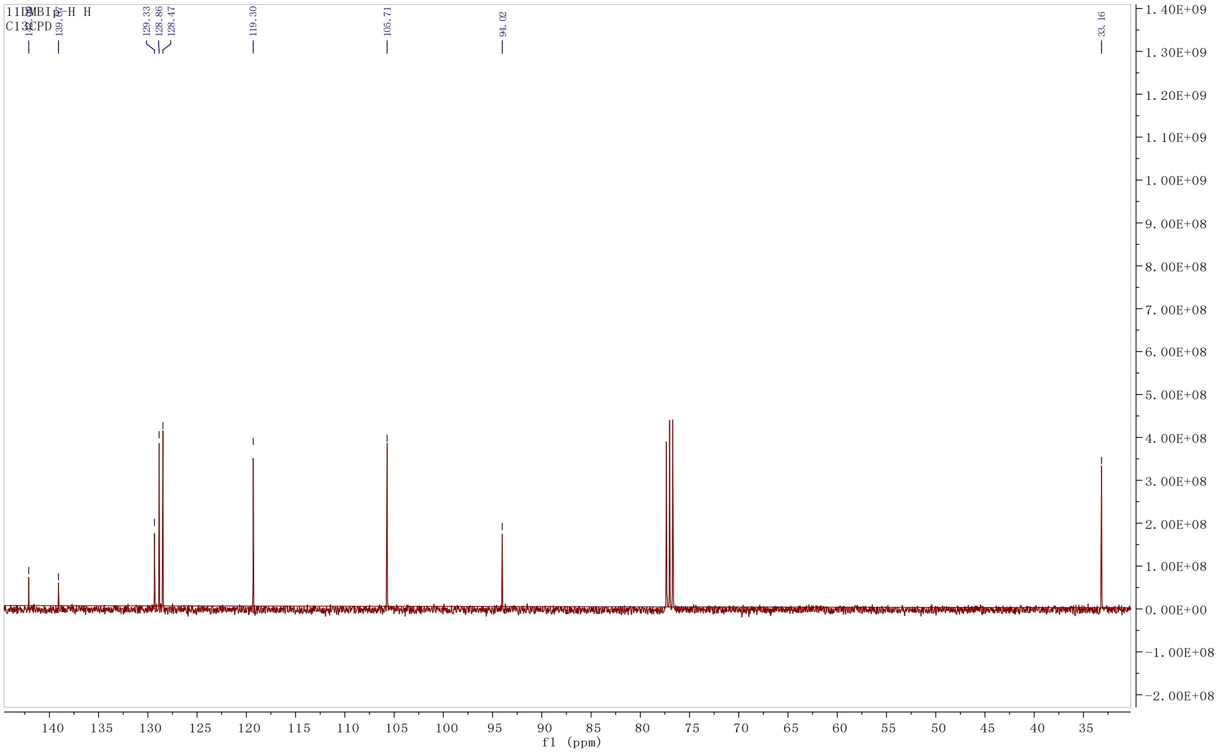

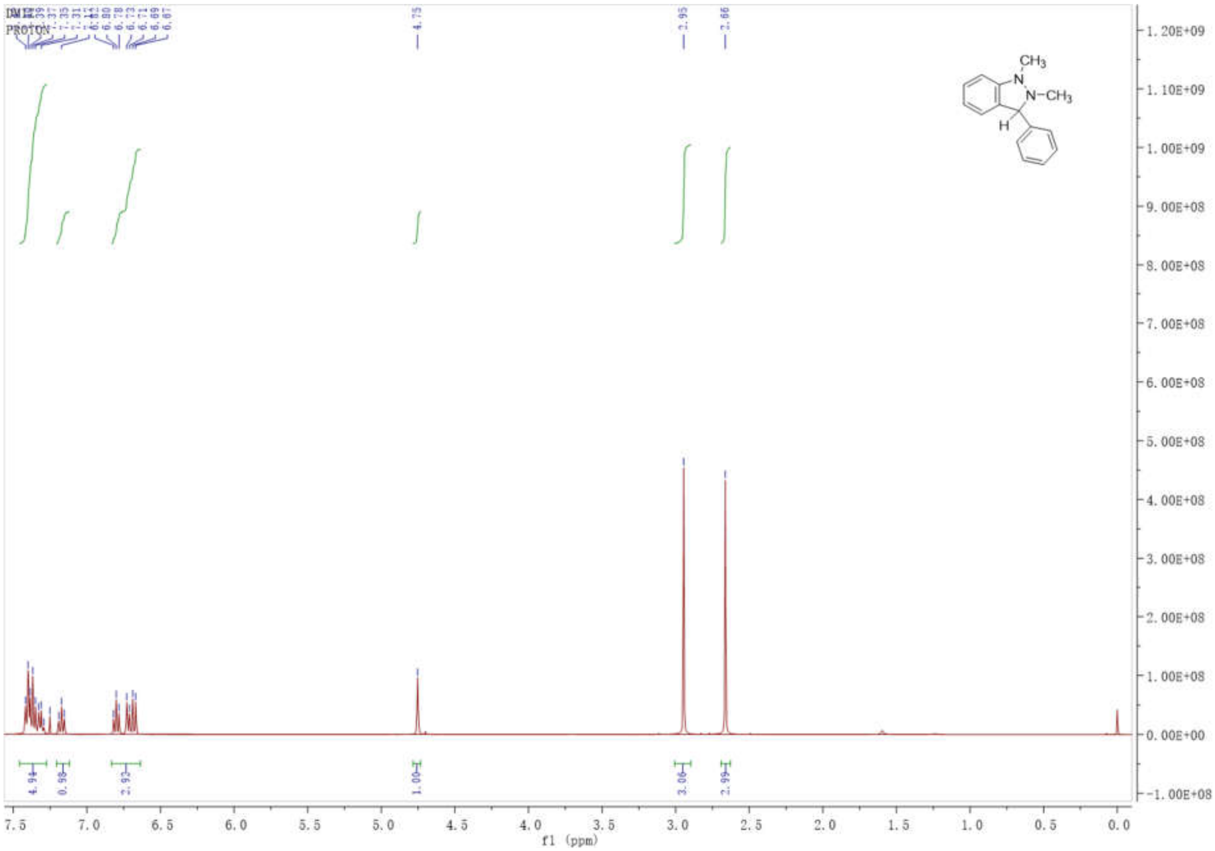

DMIZ
PROTON
4.75
2.95
2.66
CH3
N
N-CH3
H
4.94
0.98
2.93
1.00
3.06
2.99
f1 (ppm)

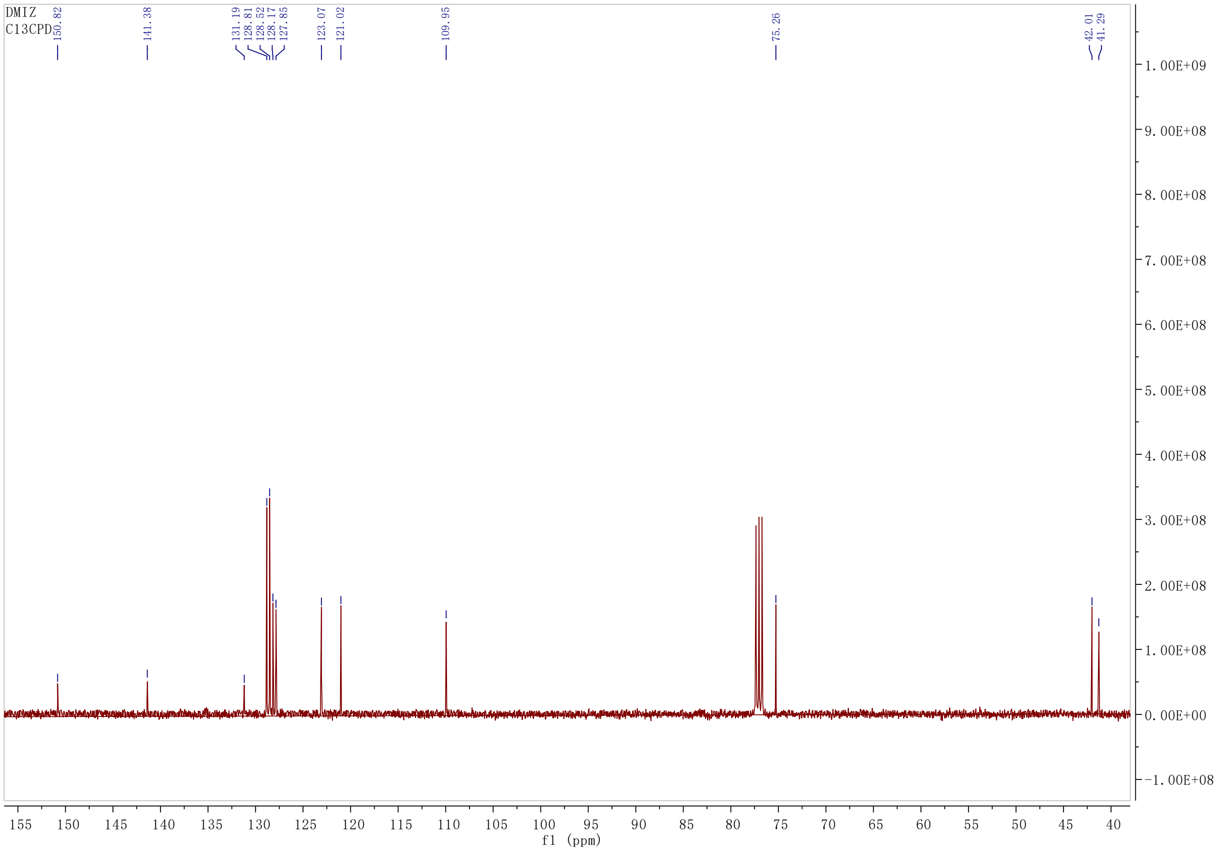

DMIZ
C13CPD
150.82
141.38
131.19
128.81
128.52
128.17
127.85
123.07
121.02
109.95
75.26
42.01
41.29
f1 (ppm)

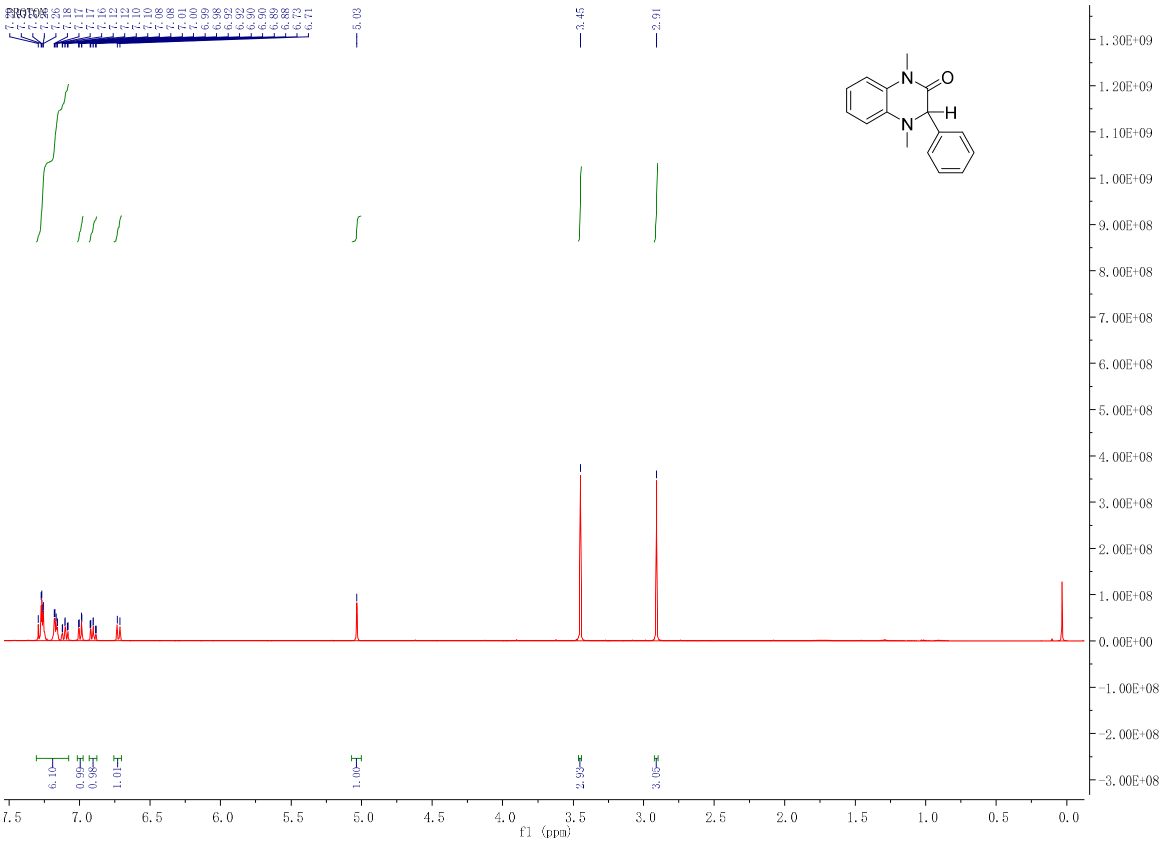
PROTON
5.03
3.45
2.91
N
O
H
N
6.10
0.99
0.98
1.01
1.00
2.93
3.05
f1 (ppm)

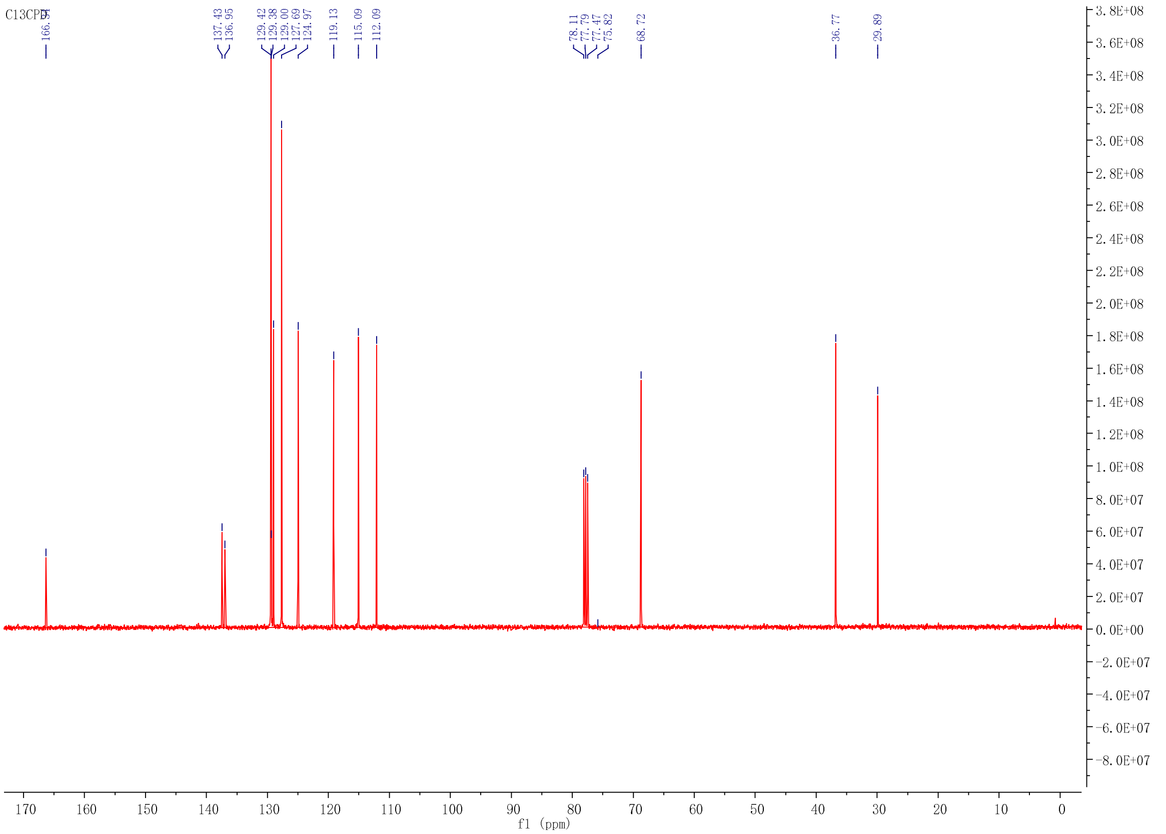
C13CPD
137.43
136.95
129.42
129.38
129.00
127.69
124.97
119.13
115.09
112.09
78.11
77.79
77.47
75.82
68.72
36.77
29.89
f1 (ppm)

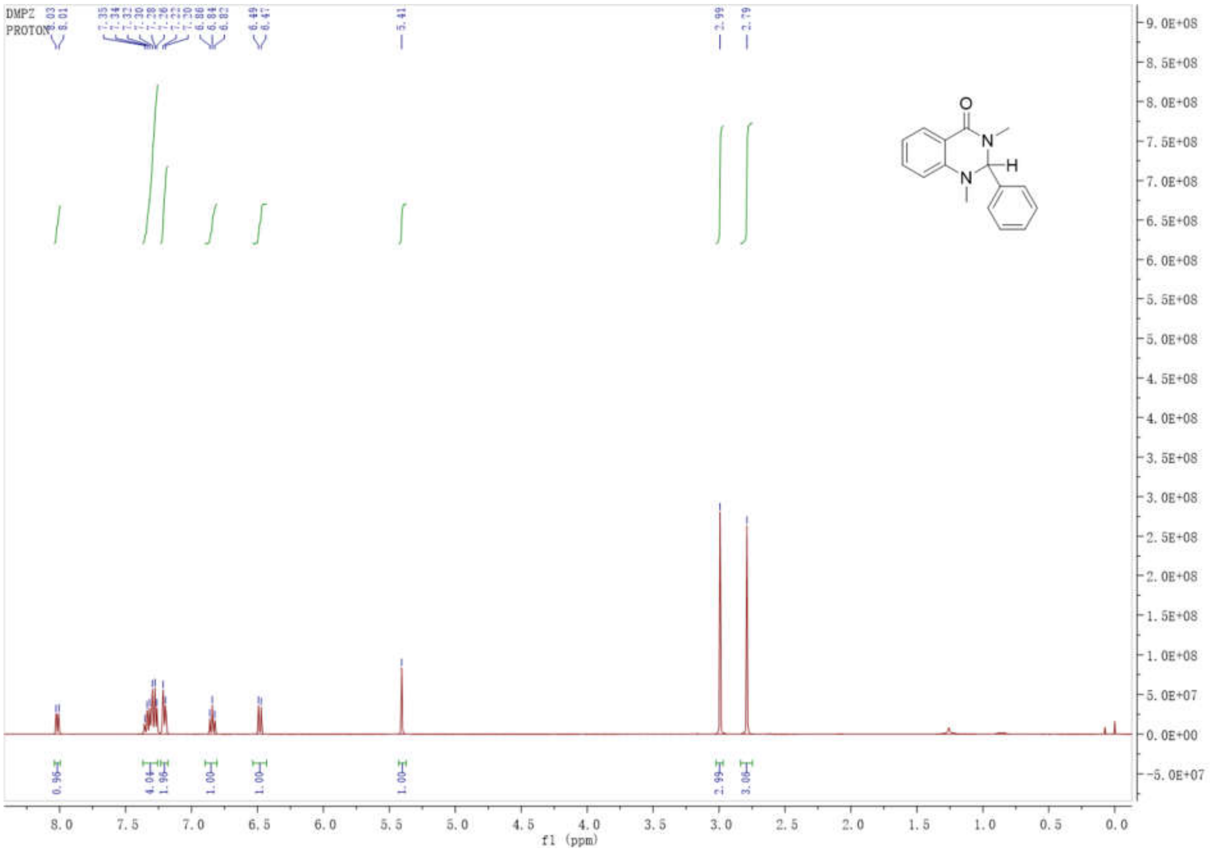
DMPZ
PROTON
8.03
8.01
7.35
7.34
7.32
7.30
7.28
7.26
7.22
7.20
6.86
6.84
6.82
6.49
6.47
5.41
2.99
2.79
0.96
4.04
1.98
1.00
1.00
1.00
2.99
3.00
O
N
N
H
f1 (ppm)

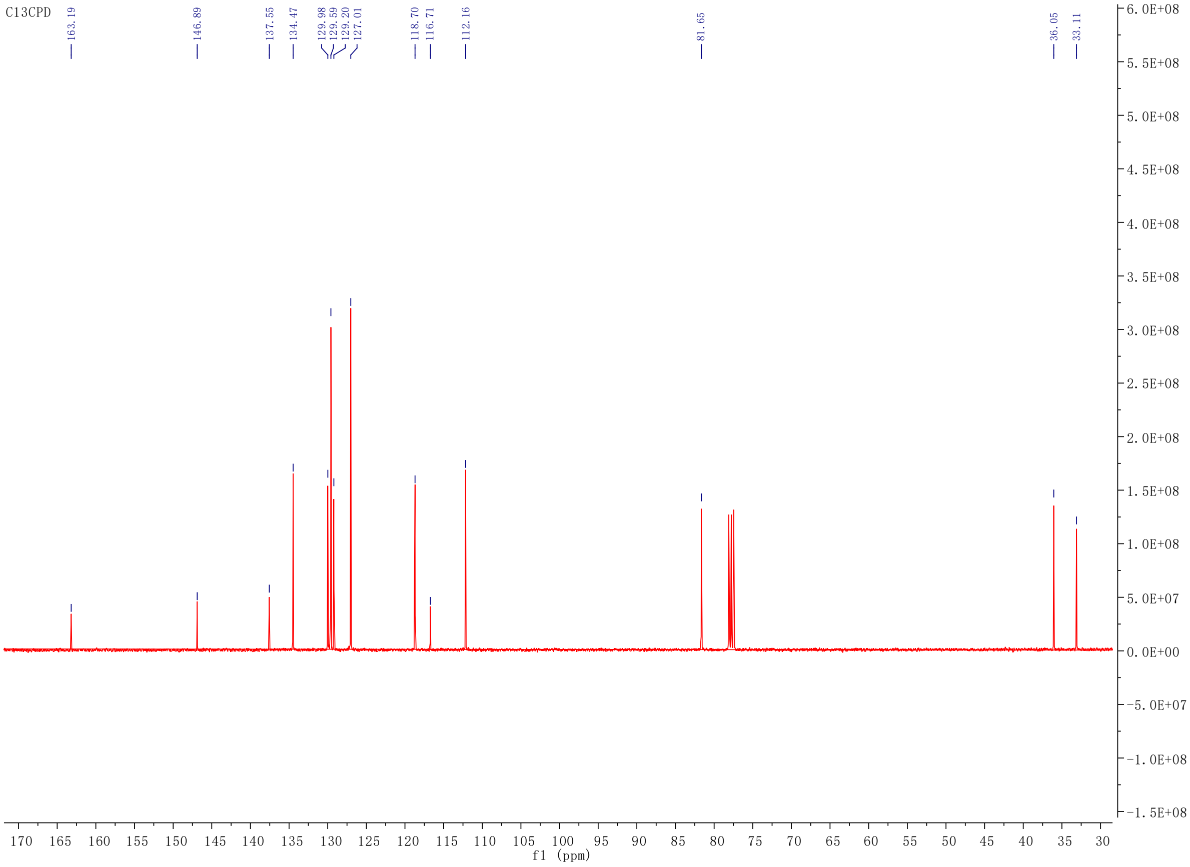
C13CPD
163.19
146.89
137.55
134.47
129.98
129.59
129.20
127.01
118.70
116.71
112.16
81.65
36.05
33.11
f1 (ppm)

SIII. The Thermodynamic and Kinetic Test Data of the Compounds with Different Substituents and the Corresponding Thermodynamic Driving Force, Kinetic Intrinsic Barrier and Thermo-kinetic Parameters Values.

In this work, we measured the hydride ion transfer rates and the corresponding molar free energy changes for a total of four groups of 12 isomeric compounds (Table S1). The thermodynamic driving force, self-exchange reaction activation energies and thermo-kinetic parameters of the corresponding compounds were calculated according to Eqs. 1-3 (Table S2). According to the analytical approach in this paper, substituent effects do not have any effect on the laws between thermodynamic driving forces, kinetic intrinsic barriers, and thermo-kinetic parameters for DMBI, DMIZ, DMPZ and, DMPX. We hope that these data will better illustrate the rationality of our approach. Therefore, these data are listed here for reference.

Table S1. Second-order rate constants (k_2), activation free energies ($\Delta G^{\neq}$), and molar free energy change ($\Delta G°$) values of oxidations of FMB and SMB isomers compounds in acetonitrile at 298K

		DMBI + $AcrH^+$	DMIZ + $AcrH^+$	DMPZ + $TEMPO^+$	DMPX + $TEMPO^+$
k_2[a]	*P* - OCH_3	3.05×10^{2}	3.84×10^{-2}	3.30	1.34×10^{2}
	P - H	1.77×10^{2}	2.17×10^{-2}	1.68	5.02×10^{1}
	P - Cl	7.54×10^{1}	10.9×10^{-2}	0.64	2.75×10^{1}
$\Delta G^{\neq}$	*P* - OCH_3	14.06	19.37	16.73	14.54
	P - H	14.38	19.71	17.14	15.13
	P - Cl	14.88	20.12	17.70	15.48
$\Delta G°$	*P* - OCH_3	-28.2	-23.3	-30.5	-24.2
	P - H	-27.0	-22.5	-27.5	-21.2
	P - Cl	-25.9	-21.7	-25.1	-18.7

Table S2. Thermodynamic driving forces [$\Delta G^{o}(XH)$], self-exchange reaction activation energies [$\Delta G^{\neq}_{XH/X}$], and thermo-kinetic parameters [$\Delta G^{\neq o}(XH)$] of DMBI, DMIZ, DMPZ, and DMPX as hydride donors in acetonitrile at 298K.

		DMBI	DMIZ	DMPZ	DMPX
$\Delta G°(XH)$	*P* - OCH_3	48.0	52.9	70.2	76.5
	P - H	49.2	53.7	73.2	79.5
	P - Cl	50.3	54.5	75.6	82.0
$\Delta G^{\neq}_{XH/X}$	*P* - OCH_3	36.44	42.16	37.52	26.84
	P - H	35.88	42.04	35.34	25.02
	P - Cl	35.78	42.06	34.06	23.22
$\Delta G^{\neq\circ}(XH)$	*P* - OCH_3	42.22	47.53	53.86	51.67
	P - H	42.54	47.87	54.27	52.26
	P - Cl	43.04	48.28	54.83	52.61
